# Parsing chronological and biological age effects on vaccine responses

**DOI:** 10.18632/aging.204572

**Published:** 2023-03-01

**Authors:** Chris P. Verschoor, George A. Kuchel

**Affiliations:** 1 Health Sciences North Research Institute, Sudbury, Ontario, Canada; 2 Northern Ontario School of Medicine, Sudbury, Ontario, Canada; 3 UConn Center on Aging, University of Connecticut School of Medicine, Farmington, CT 06032, USA

**Keywords:** biological age, frailty, vaccination, influenza

As illustrated by the COVID-19 pandemic, older age, particularly when accompanied by common chronic illnesses of aging, is arguably the most significant population attributable factor for severe outcomes of acute respiratory infection, including the risk of hospitalization, disability and death. In the absence of widely available and highly effective treatments, vaccines remain our most powerful tool to help overcome this vulnerability through the prevention of primary infection, and far more importantly, by improving clinical outcomes once infection does take place. In the case of SARS-CoV-2, vaccine effectiveness (VE) against hospitalization was remarkable for dominant strains prior to omicron, whereas for influenza or *Streptococcus pneumoniae* VE ranges from 80% to <10%, depending on the season and infecting strain/serotype. Nonetheless, for all three pathogens VE decreases with age, which is caused by deficiencies in the capacity of older adults’ immune systems to mount productive and persistent antibody and/or cell-mediated responses to the vaccine [1]. Given that extremely large, costly and typically lengthy clinical trials are often required to estimate VE reliably, the vast majority of human vaccine studies assess immune correlates of protection as a proxy to VE. For these studies, antibody-related parameters such as neutralization capacity are most commonly employed since they are generally simpler from a technical standpoint and many have been rigorously standardized.

Two FDA-approved seasonal influenza vaccines have been developed to overcome aging-related declines in immune responses, one employing an adjuvant (Fluad, Seqirus) and the other increased dosage (Fluzone High Dose, Sanofi Pasteur). Both vaccines are safe and improve VE and vaccine immunogenicity to a greater extent than earlier non-adjuvanted or standard dose formulations; however, they are also associated with higher rates of non-fatal adverse events and can cost upwards of three times that of standard formulations. Considering that many adults above 65 maintain the immunological fitness required to generate protective immunity from standard vaccine formulations, utilizing a “blanket” approach where all older adults at a specified age are provided these improved vaccines may not be optimal. However, a question remains: how do we identify those vulnerable individuals who require the additional protection of an enhanced vaccine platform?

Although informative, cross-sectional studies comparing immune parameters across age groups to understand “immune aging” risk ignore the degree to which departures from healthy aging might contribute. Healthy aging can be considered a health trajectory where sufficient function is maintained, thereby enabling optimal well-being until end of life; hence, significant deviations from this trajectory will drive vulnerability to adverse outcomes beyond what can attributed to chronological age alone. To date, most studies evaluating such deviations from normative aging do so by enumerating a defined set of clinical manifestations of the frailty syndrome, or a more cross-cutting set of health deficits that comprise a frailty index [2]. Although frailty is clearly associated with an increased risk of developing severe illness with poor outcomes when infected with influenza and SARS-CoV-2 viruses, specific mechanistic underpinnings behind this vulnerability remain understudied. As posited by the geroscience hypothesis [3], it is likely that these mechanisms are woven amongst the vast cellular and molecular networks that promote and are disrupted by aging and frailty, and is best reflected by a construct commonly referred to as biological age. Quantified using sets of biomarkers (e.g. circulating proteins or metabolites, cellular or gene transcript abundance, and DNA methylation levels) that are conceptually or empirically associated with the aging process, biological age represents the overall integrity of one’s physiological systems and their resilience to pathological stress. It is normally expressed relative to chronological age, where estimates greater than zero would suggest increased deviation from a healthy aging trajectory and a greater risk of physiological dysfunction in the face of an exogenous stressor. Recently, our group published one of the first studies examining the association between biological age and neutralizing antibody responses of adults 65 and older to the seasonal influenza vaccine over four consecutive seasons [4]. We quantified biological age using a commonly employed algorithm facilitated through publicly available software developed by the Belsky research group (Columbia University Mailman School of Public Health), which estimates the deviation of an age-variant panel of blood biomarkers for a given individual from what is observed in the population. As with previous studies of frailty [5], we observed little correlation for standard vaccine formulation recipients. In contrast, for high dose recipients, antibody responses were significantly *higher* in those with an older or accelerated biological age [4]. The effect sizes of these associations were as much as twice what we previously estimated for frailty [5], and causal mediation analysis suggested a pathway prominently featuring chronic inflammation and possibly CD57-expressing natural killer (NK) cells [6].

On one level, our findings demonstrating enhanced humoral responses in individuals with evidence of more rapid biological aging may seem counterintuitive, given that VE decreases with chronological age and frailty. However, a careful evaluation of immune aging through a systems-based lens, one that highlights the dynamic and temporal nature of immune responses [1], sheds a far more nuanced and ultimately insightful perspective on our observations. For example, it is important to consider that imbalances in Th2 vs. Th1 immunity that have been demonstrated by previous literature on frail older adults may result in enhanced humoral responses in the context of accelerated biological age. It is also possible that the high-dose vaccine may be unable to overcome inherent chronological/biological age-related defects in CD8 T-cell function and narrowing diversity towards what is commonly described as an “exhausted” phenotype. Thus, the additional antigenic pressure is left only to boost antibody-mediated mechanisms, which during secondary exposures can be elicited by DC/T-dependent and -independent as well as lymph node macrophage and NK-cell mediated pathways. Some of these pathways have been shown to be dependant on pro-inflammatory cytokines such as TNF [7] and IL-6 [8], particularly in the context of aging.

Ultimately, while both chronological and biological age appear to be important determinants of vaccine-preventable outcomes in older adults, the underlying context and mechanisms of their effects remain unclear. Do deviations from a healthy aging trajectory merely exacerbate age-related defects in vaccine-induced immune mechanisms, aligning with the hypothesis that these individuals are experiencing a form of “accelerated aging”? Alternatively, do these deviations comprise independent cellular and molecular alterations that impair vaccine responses, or in the case of our recent work [4], improve certain aspects of it? Another important consideration is the manner by which we quantify biological aging. Numerous approaches exist that may differ both methodologically and conceptually, and as such, are believed to embody different aspects of biological aging and the underlying systems that they comprise. This will likely be of great value as discrepant associations amongst these approaches with vaccine induced immunity, the context of which also depends heavily on the pathogen/illness and vaccine platform in consideration, may offer clues to those immunological systems that have been disrupted by biological aging. To answer these many questions, we envision a need for new VE studies combining the use of single cell immunogenomics with systems-based analysis involving sampling at multiple timepoints from older adults who have undergone careful clinical and biological phenotyping. This will provide cellular and molecular immunologists adequate evidence to initiate in-depth investigations into potential pathways or molecules that can be targeted to improve vaccines.
